# Improvement of acute phase symptoms of pemphigus foliaceus with spesolimab

**DOI:** 10.1016/j.jdcr.2024.08.035

**Published:** 2024-09-18

**Authors:** Shohei Iida, Ken Muramatsu, Ayaka Mizuno, Akinobu Hayashi, Yuki Mizutani, Hideyuki Ujiie, Kazumitsu Sugiura, Keiichi Yamanaka

**Affiliations:** a Department of Dermatology, Graduate School of Medicine, Mie University, Tsu, Mie, Japan; b Department of Dermatology, Hokkaido University Graduate School of Medicine, Sapporo, Hokkaido, Japan; c Department of Dermatology, Yokkaichi Municipal Hospital, Yokkaichi, Mie, Japan; d Department of Oncologic Pathology, Graduate School of Medicine, Mie University, Tsu, Mie, Japan; e Department of Dermatology, Fujita Health University, Toyoake, Aichi, Japan

**Keywords:** erythema, inflammatory reaction, pemphigus foliaceus, pustule, spesolimab

## Introduction

The inflammatory cytokine interleukin (IL)-36 is expressed in a variety of organs and cell types, including skin, brain, joints, and synovial tissue, and it plays a role in antigen presentation, immune cell activation, and proinflammatory pathways.^1^, ^2^, ^3^, ^4^ Dysregulation of IL-36 has been implicated in the pathogenesis of inflammatory diseases, such as generalized pustular psoriasis (GPP), psoriasis vulgaris, pyoderma gangrenosum, and atopic dermatitis. During inflammation, IL-36 cytokines are enhanced and expressed by keratinocytes, epithelial cells, and inflammatory monocytes/macrophages.^5^ The accumulating evidence suggests that IL-36 may be an inducer of innate immunity. The administration of humanized IgG1 antagonistic anti-IL-36 receptor monoclonal antibody, spesolimab, is effective for GPP flare and is widely used to treat the acute phase of GPP. Here, we report a case of pemphigus foliaceus (PF) showing marked improvement in acute phase symptoms with its use.

## Case report

A 65-year-old woman presented with numerous pustule formations and annular erythematous lesions that had appeared from her trunk and spread to the whole body 5 months prior. Previous treatments with fexofenadine hydrochloride and topical difluprednate had been ineffective. Her medical and family history was not specific.

**Fig 1:**
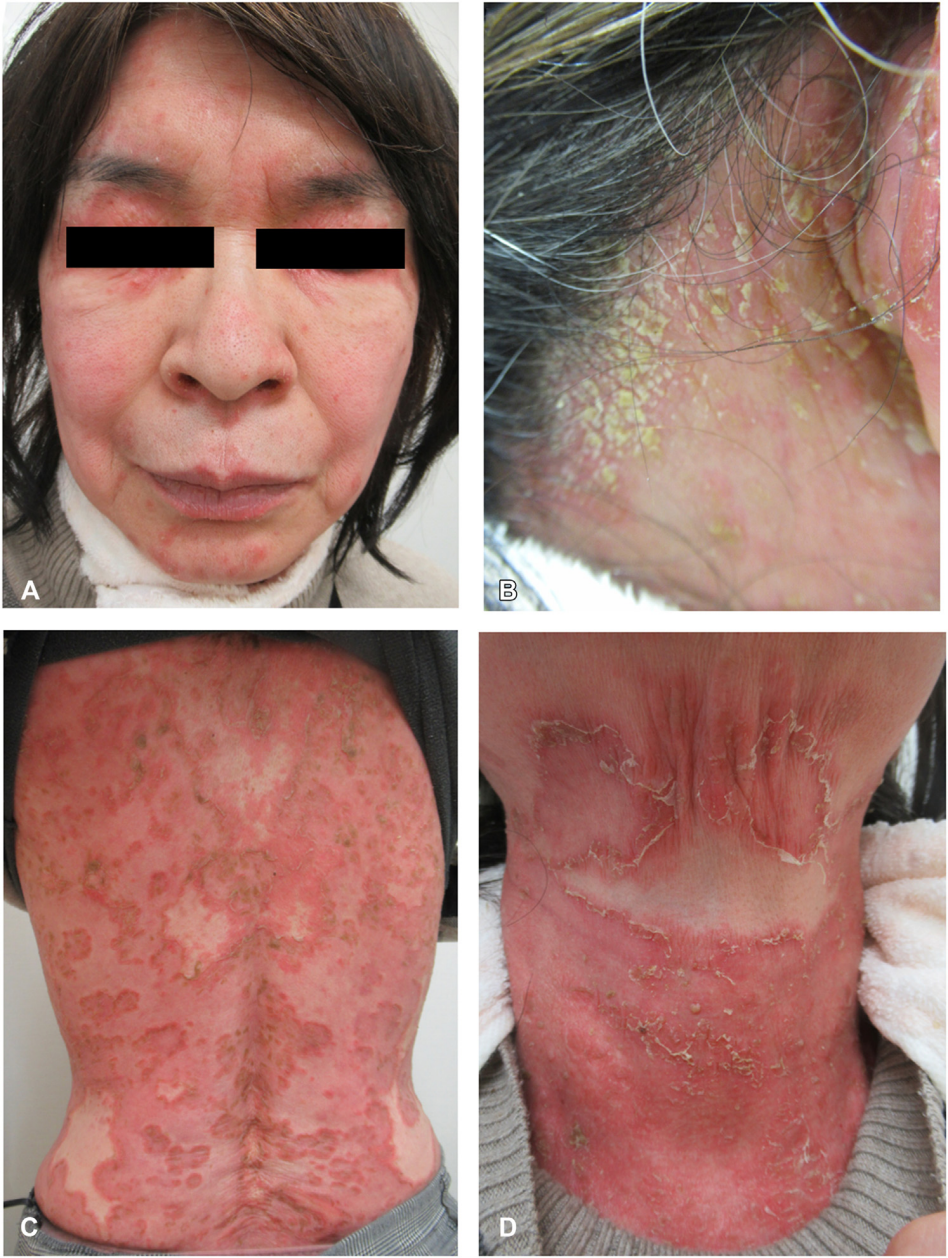
**A-D,** Clinical features at the first visit. A 65-year-old woman presented with numerous pustule formations and annular erythematous lesions that had appeared from her trunk and spread to the whole body. Numerous well-defined red annular plaques ranging from 5 to 30 mm in diameter are on her head, face, neck, and forearms. Pustules were fragile and clusters were detected in the neck and head.

**Fig 2:**
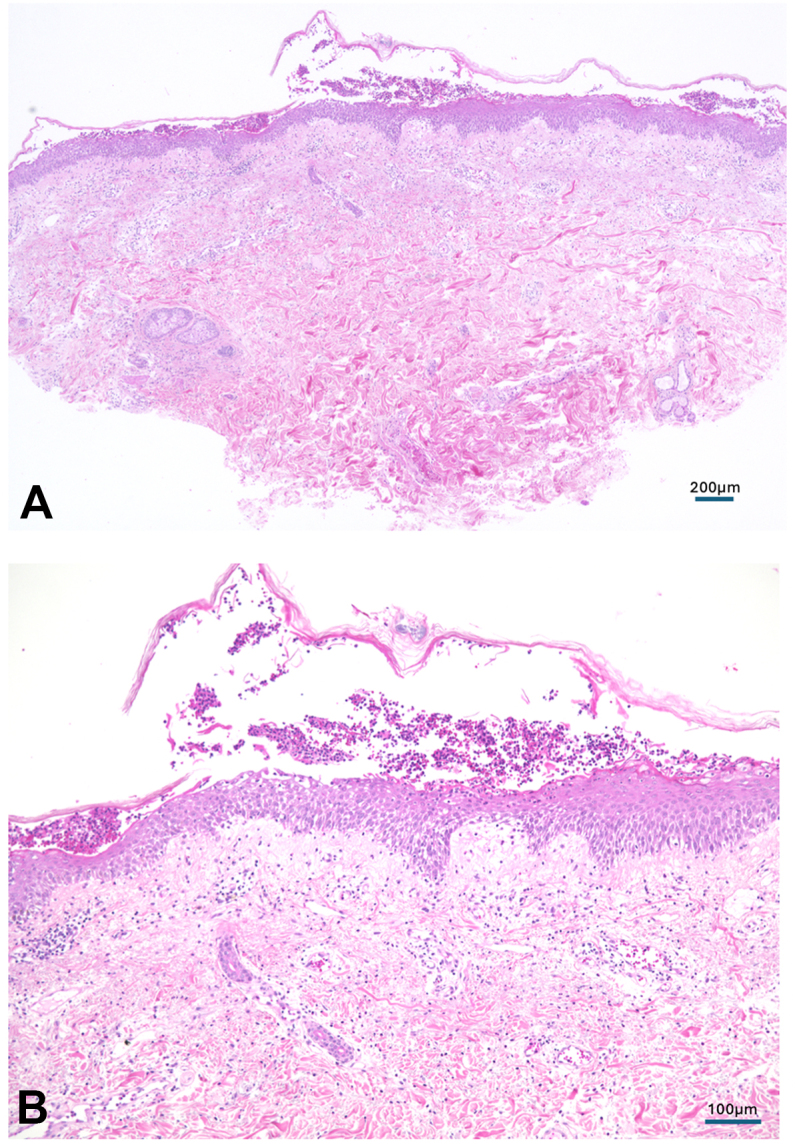
A skin biopsy from erythema including pustules on upper portion of the arms showed subcorneal pustule formation, spongiotic changes, and vacuolar degeneration were present in the epidermis. In the dermis, a mild to moderate perivascular inflammatory cell infiltrate with mixed lymphocytes and neutrophils was observed in the predominant perivascular zone in the papillary dermis. (**A, B,** Hematoxylin-eosin stain.)

Upon cutaneous examination, we observed numerous well-defined red annular plaques ranging from 5 to 30 mm in diameter on her head, face, neck, and forearms. Fragile pustules and clusters were detected in the neck and head (Fig 1, *A*-*D*). Low-grade fever and general fatigue were recognized. The pustules were sterile. A skin biopsy revealed subcorneal pustule formation, spongiotic changes, and vacuolar degeneration in the epidermis. A mild to moderate inflammatory cell infiltrate with mixed lymphocytes and neutrophils is observed in the predominant perivascular zone in the papillary dermis (Fig 2, *A*, *B*). Blood tests showed leukocytosis with neutrophilia (9250/μL and 8320/μL, respectively). The serum albumin level was lowered at 3.42 g/dL (4.1-5.1), and slightly elevated C-reactive protein at 0.19 mg/dL (–0.14) and erythrocyte sedimentation rate at 21 mm/h (3-15), and a negative antinuclear antibody result was detected. There was no arthritis or uveitis. There were no noted symptoms in other organs.

**Fig 3:**
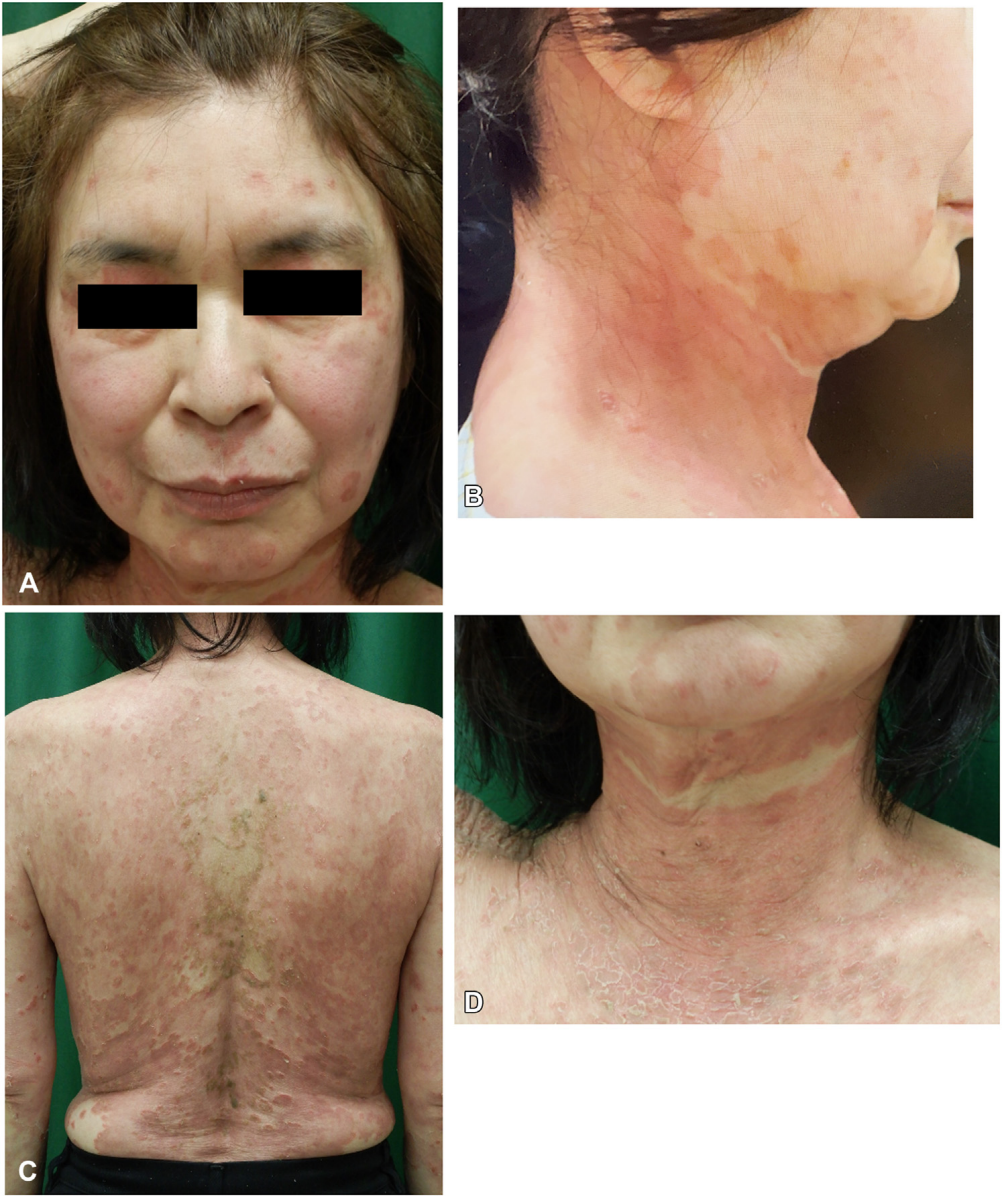
Clinical features a week after starting intravenous spesolimab. **A-D**, Systemic symptoms, pustule, and erythema showed marked improvement within 10 days.

**Fig 4:**
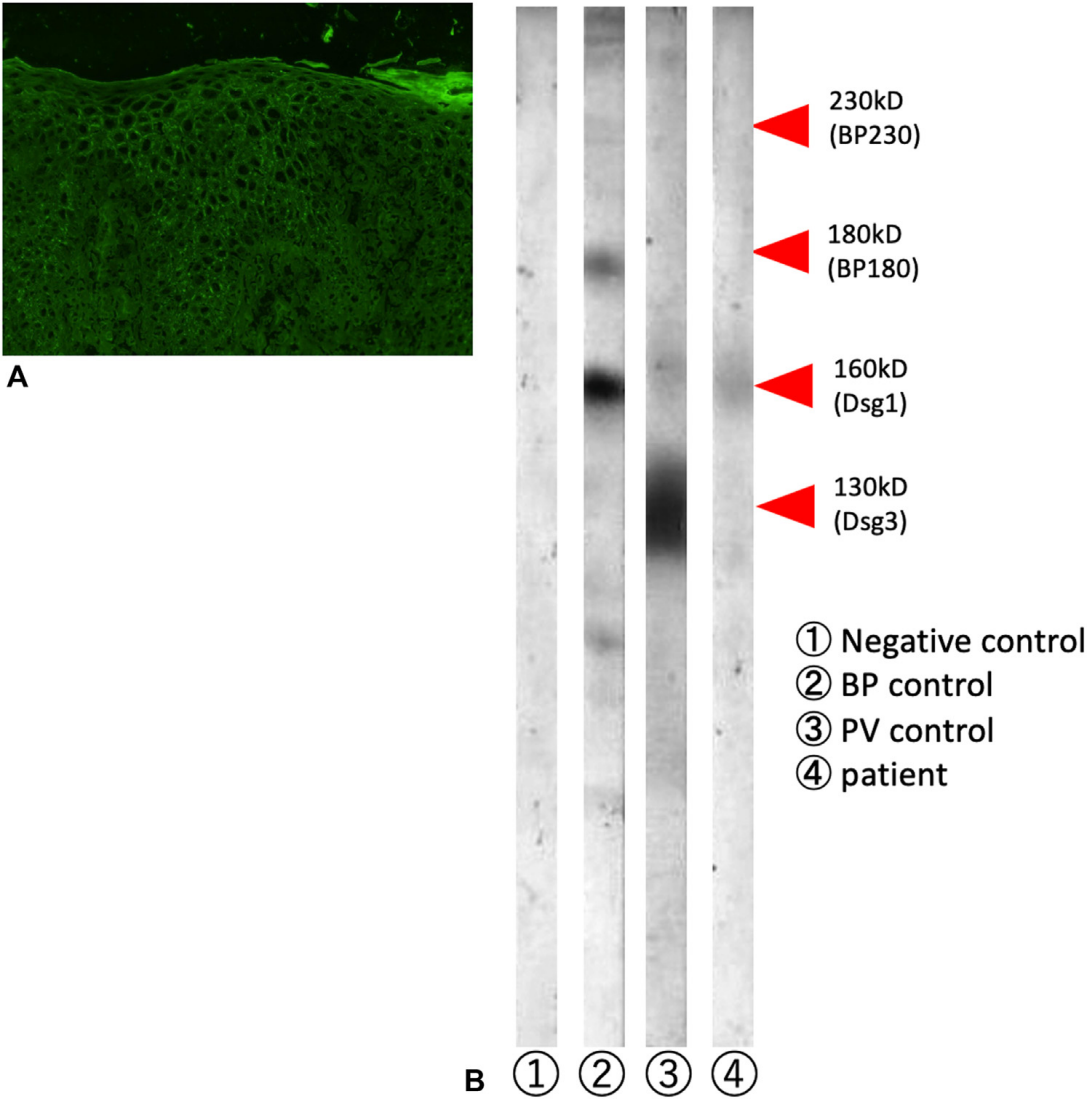
Direct immunofluorescence of the skin demonstrated deposits of IgG and C3 in the upper layer of the epidermis but not of IgM or IgA in intercellular spaces of the epithelia (**A**). Western blotting analysis was performed using epidermal extracts as substrates. Patient serum was diluted 20-fold, and a 100-fold dilution of antipatient serum was used as a secondary antibody. A 160 kb band corresponding to desmoglein 1 was observed. Lane 1 was negative control, lane 2 was serum from a patient with bullous pemphigoid, and lane 3 was serum from a patient with pemphigus vulgaris (**B**).

Acute-phase aseptic pustular formation and erythema are considered a GPP based on pathologic findings. A 900 mg dose of intravenous spesolimab, targeting the IL-36 receptor, was administrated to suppress systemic symptoms (day 0), followed by a second dose after 1 week for persistent flare symptoms (day 7). Low-grade fever and general fatigue, pustule, and erythema markedly improved (day 10, Fig 3, *A*-*D*). However, further blood examination revealed elevated desmoglein 1 (5660.0 U/mL, –20), but desmoglein 3 was negative. Direct skin fluorescent immunostaining results revealed intercellular IgG and C3 deposition, but not of IgM or IgA in the upper layers of the epidermis (Fig 4, *A*). Indirect fluorescence using normal human skin detected total IgG (titer 1/160) autoantibodies against intracellular spaces. Western blotting analysis using epidermal extract as substrate showed the upbeat band at 160 kb for the patient’s plasma (Fig 4, *B*). Results of IL36RN, which encodes IL-36 receptor antagonist, whole coding sequencing and caspase recruitment domain family member 14 Ex2-E4 direct sequencing showed no mutations.

These findings confirmed a diagnosis of PF. Although no new aseptic pustules developed, a very high Des1 antibody titer and persistent erythema with induration warranted additional treatment. Consequently, oral prednisolone was administered at 60 mg daily (1 mg/kg of body weight) starting from day 30. Since then, erythema with induration has improved, and the dosage of the oral steroid has been steadily reduced from day 50 without relapse.

## Discussion

IL-36 is crucial in maintaining a healthy state and preventing hyper-inflammation in tissues,^4^ and also is a key regulator and an inducer of autoimmunity^6^ and, possibly, works as an alarmin. Activated IL-36α/β/γ bind the IL-36R and recruit IL-1RAcP, bringing together the Toll/IL-1R domains of the 2 heterodimeric receptors and causing an intracellular signaling cascade that involves mitogen-activated protein kinases, signal transducer and activator of transcription 3, and nuclear factor kappa B-dependent transcription.^4^^,^^6^ The binding of IL-36 to the IL-36R leads to the intracellular production of more IL-36, IL-1β, tumor necrosis factor-alfa, T helper cells-, and neutrophil-attracting chemokines, enhancing inflammation.^7^^,^^8^ PF is a chronic autoimmune skin disorder characterized by subcorneal acantholytic bullae and the deposition of IgG1 and IgG4 antibodies on desmoglein 1and desmoglein 3. However, atypical cases of PF have been reported, presenting with neutrophilic pustules, which complicates the differential diagnosis with GPP.^9^^,^^10^ On the other hand, GPP is a systemic inflammatory disease characterized by erythema and sterile, neutrophil-rich pustules. Pathologically, it is defined by the presence of Kogoj spongiform pustules. Alterations in the expression of various components of the IL-36 pathway have been shown to initiate a positive feedback loop of uncontrolled signaling, leading to excessive production of inflammatory cytokines. This cascade further results in the induction of chemokines and the recruitment of neutrophils to the epidermis. Therefore, it is possible that IL-36, such as GPP, mobilizes neutrophils to the epidermis and aggravates the disease in PF. In using spesolimab, careful observation is required because serious infections including tuberculosis, viruses, bacteria, etc have been reported. When the drug is used for diseases for which it is not indicated, more careful follow-up is required because there is no accumulation of adverse reactions. However, this is strongly suggested by the fact that spesolimab treatment in the current case improved general and skin symptoms, although IL-36RN mutation was undetected, suggesting upstream signaling of IL-36 is enhanced in the current case. Long-term follow-up is necessary for the relapse of PF, but this report demonstrates the significant effect of spesolimab on the acute symptoms of PF.

## Conflicts of interest

None disclosed.
